# Current and emerging evidence-based treatment options in chronic migraine: a narrative review

**DOI:** 10.1186/s10194-019-1038-4

**Published:** 2019-08-30

**Authors:** Elio Clemente Agostoni, Piero Barbanti, Paolo Calabresi, Bruno Colombo, Pietro Cortelli, Fabio Frediani, Pietrangelo Geppetti, Licia Grazzi, Massimo Leone, Paolo Martelletti, Luigi Alberto Pini, Maria Pia Prudenzano, Paola Sarchielli, Gioacchino Tedeschi, Antonio Russo, The Italian chronic migraine group

**Affiliations:** 1 S.C. Neurologia e Stroke Unit ASST Grande Ospedale Metropolitano Niguarda, Milan, Italy; 2 Headache and Pain Unit, Department of Neurological, Motor and Sensorial Sciences, IRCCS San Raffaele Pisana, Rome, Italy; 3 San Raffaele University, Rome, Italy; 4 Neurologic Clinic, Ospedale Santa Maria della, Perugia, Italy; 5 Dipartimento di Neurologia, Università Vita-Salute, Ospedale San Raffaele, Milan, Italy; 6 IRCCS- Istituto di Scienze Neurologiche di Bologna, Bologna, Italy; 7 DIBINEM- University of Bologna, Bologna, Italy; 8 Headache Centre, UOC Neurologia e Stroke Unit, P.O. San Carlo Borromeo, ASST Santi Paolo e Carlo, Milan, Italy; 9 Department of Health Sciences, Section of Clinical Pharmacology and Headache Center, University of Florence, Florence, Italy; 10 Fondazione IRCCS Istituto Neurologico “C.Besta”, Milan, Italy; 11 Department of Clinical and Molecular Medicine, Sapienza University, Rome, Italy; 12 Headache Center, Department of Biomedical, Metabolic and Neuro Science, University of Modena and Reggio Emilia, Modena, Italy; 13 Headache Center, Department of Basic Medical Sciences, Neurosciences and Sense Organs, University of Bari, Bari, Italy; 14 Headache Center, Neurologic Clinic, Ospedale Santa Maria della Misericordia, Perugia, Italy; 15 Headache Center Department of Medical, Surgical, Neurological, Metabolic, and Aging Sciences, University of Campania “Luigi Vanvitelli”, Naples, Italy

**Keywords:** Chronic migraine, Fremanezumab, onabotulinumtoxinA, Prophylaxis, Topiramate, Anti-CGRP monoclonal antibodies

## Abstract

**Background:**

Chronic migraine is a disabling condition that is currently underdiagnosed and undertreated. In this narrative review, we discuss the future of chronic migraine management in relation to recent progress in evidence-based pharmacological treatment.

**Findings:**

Patients with chronic migraine require prophylactic therapy to reduce the frequency of migraine attacks, but the only currently available evidence-based prophylactic treatment options for chronic migraine are topiramate and onabotulinumtoxinA. Improved prophylactic therapy is needed to reduce the high burden of chronic migraine in Italy. Monoclonal antibodies that target the calcitonin gene-related peptide (CGRP) pathway of migraine pathogenesis have been specifically developed for the prophylactic treatment of chronic migraine. These anti-CGRP/R monoclonal antibodies have demonstrated good efficacy and excellent tolerability in phase II and III clinical trials, and offer new hope to patients who are currently not taking any prophylactic therapy or not benefitting from their current treatment.

**Conclusions:**

Treatment of chronic migraine is a dynamic and rapidly advancing area of research. New developments in this field have the potential to improve the diagnosis and provide more individualised treatments for this condition. Establishing a culture of prevention is essential for reducing the personal, social and economic burden of chronic migraine.

## Background

Chronic migraine (CM), defined by the current International Headache Society classification of headache disorders (ICHD-3) as headache occurring on ≥15 days/month for > 3 months with features of migraine on ≥8 days/month [1], is a disabling condition that affects 0.5% to 5% of the general population [2, 3]. However, the true prevalence of CM is difficult to estimate because of heterogeneous data collection instruments, differences in diagnostic strategies between headache centres, patient recall bias, and the potential for patients to overestimate headache frequency, especially if they have psychiatric comorbidities. Compared with episodic migraine (EM), CM is less common but is associated with greater headache-related disability, higher impact on physical, social and occupational functioning, and worse health-related quality of life [2, 4, 5]. Patients with CM also have an increased incidence of co-morbid psychiatric and medical conditions [6, 7], resulting in complex cases of chronic multidimensional migraine. Despite the considerable individual and societal consequences of CM, it remains an underdiagnosed and undertreated condition worldwide [2, 8], and Italy is no exception [9, 10].

Migraine has been conceptualised as a continuum that ranges from EM to CM, with variations in headache days per month and symptoms [11]. About 3% of patients with EM progress to CM each year [11–13], but there is a natural within-patient variation in headache-day frequency, meaning that patients can fluctuate between EM and CM [14]. This natural fluctuation needs to be considered when clinicians diagnose and treat CM [14]. Accompanying symptoms of CM can include nausea, vomiting, photophobia, phonophobia and osmophobia, but nausea, vomiting, photophobia and phonophobia are often less pronounced with CM than with EM [15].

The mechanisms underlying the progression of EM to CM are complex and not fully understood; however, modifiable risk factors for progression include the frequency of headache attacks, overuse of acute migraine medication, ineffective acute treatment, stressful life events and obesity [8, 12, 16, 17]. Medication-overuse headache (MOH) is now considered a sequela rather than a cause of migraine and can co-exist with CM [1, 18, 19]. In addition to risk factor modification, and the appropriate and effective acute treatment of migraine, all patients with CM need prophylactic treatment to reduce the headache frequency, severity and associated disability [8, 20]. However, low proportions of patients who are candidates for prophylactic treatment actually receive it [8]. Within Europe, prophylactic treatment appears to be particularly underused in Italy [10].

This review summarises strategies for the prophylactic treatment of CM, and highlights the importance of creating a culture for the timely prevention of CM.

### Search methods

As this is a narrative review, we did not conduct a systematic literature search. However, a search of the PubMed database was conducted in May 2018, with no date limits, using the search terms “chronic migraine” and “treatment”, and the results were screened for relevance to the review topic. Articles were also added based on the authors’ knowledge of the area.

## Understanding the pathophysiology of chronic migraine

The pathophysiology of CM is not fully understood, but there is evidence to indicate that functional changes occur in the brains of patients with CM, including increased cortical hyperexcitability, central trigemino-thalamic sensitisation and defective descending pain modulatory activity [21–23]. It is postulated that recurring migraine episodes and comorbid conditions, such as medication overuse or anxiety/depression, may lead to dysfunction of pain modulation pathways, with reduced nociceptive thresholds and atypical release of nociceptive molecules [11, 22]. This may cause increasing central sensitisation of the trigeminal and thalamic neurons, with little recovery between attacks, leading to progression from EM to CM [22, 24]. Cutaneous allodynia and increased activation of the trigeminovascular pathway, both of which occur in migraine [25, 26], implicate hyperexcitability of certain central nervous system structures and increased release of nociceptive neuropeptides, such as calcitonin gene-related peptide (CGRP), which is highly expressed in trigeminal neurons in the central and peripheral nervous system [11, 21, 24, 27]. The crucial role of CGRP in headache pain notwithstanding, the first step of the migraine attack is likely to involve central mechanisms, as suggested by functional neuroimaging findings of a hypothalamic involvement during the early attack stages [28], as well as the concept that migraine may occur without pain in the paediatric population (migraine-associated periodic syndromes) and in patients who experience migraine with aura.

CGRP, which is involved in pain modulation, perception and sensitisation, seems to have a major role in the pathogenesis of migraine {Goldberg, 2015 #47;Ho, 2010 #21;Edvinsson, 2019 #139}. Activation of transient receptor potential (TRP) channels, which coexist with CGRP in the same nociceptive neurons, promotes excitation of the trigeminovascular pathway, release of CGRP and pain [29–31]. At the central neuronal level, release of CGRP is thought to contribute to cortical spreading depression, which is a key pathophysiological component of migraine with aura [32, 33]. Release of CGRP from peripheral trigeminal fibres is also believed to cause vasodilation and mast cell degranulation, resulting in a persistent pro-inflammatory sensitisation inducing trigeminal nociceptors sensitisation [27]. Interictal levels of CGRP in peripheral blood are higher in patients with CM than EM [34], suggesting altered interictal activity of the trigeminal nervous system in CM [35].

## Treatment of chronic migraine

Some patients with low frequency EM can be managed with effective acute therapy (i.e. drugs taken during the prodrome or the migraine attack to abort it) without prophylactic treatment, but patients with CM invariably require prophylactic treatment [8, 36]. Whereas the goal of acute therapy is to abort a migraine attack once it has started, the goals of prophylactic treatment are to prevent attacks, thereby reducing headache frequency, severity and associated disability and decreasing reliance on acute treatment, which may be contributing to concurrent MOH [8, 20]. An additional goal may be to prevent progression of EM to CM in patients with high frequency EM [8]. Patients with EM can be treated with recommended prophylactic therapy based on the number of attacks > 4 and the degree of disability.

**Fig. 1:**
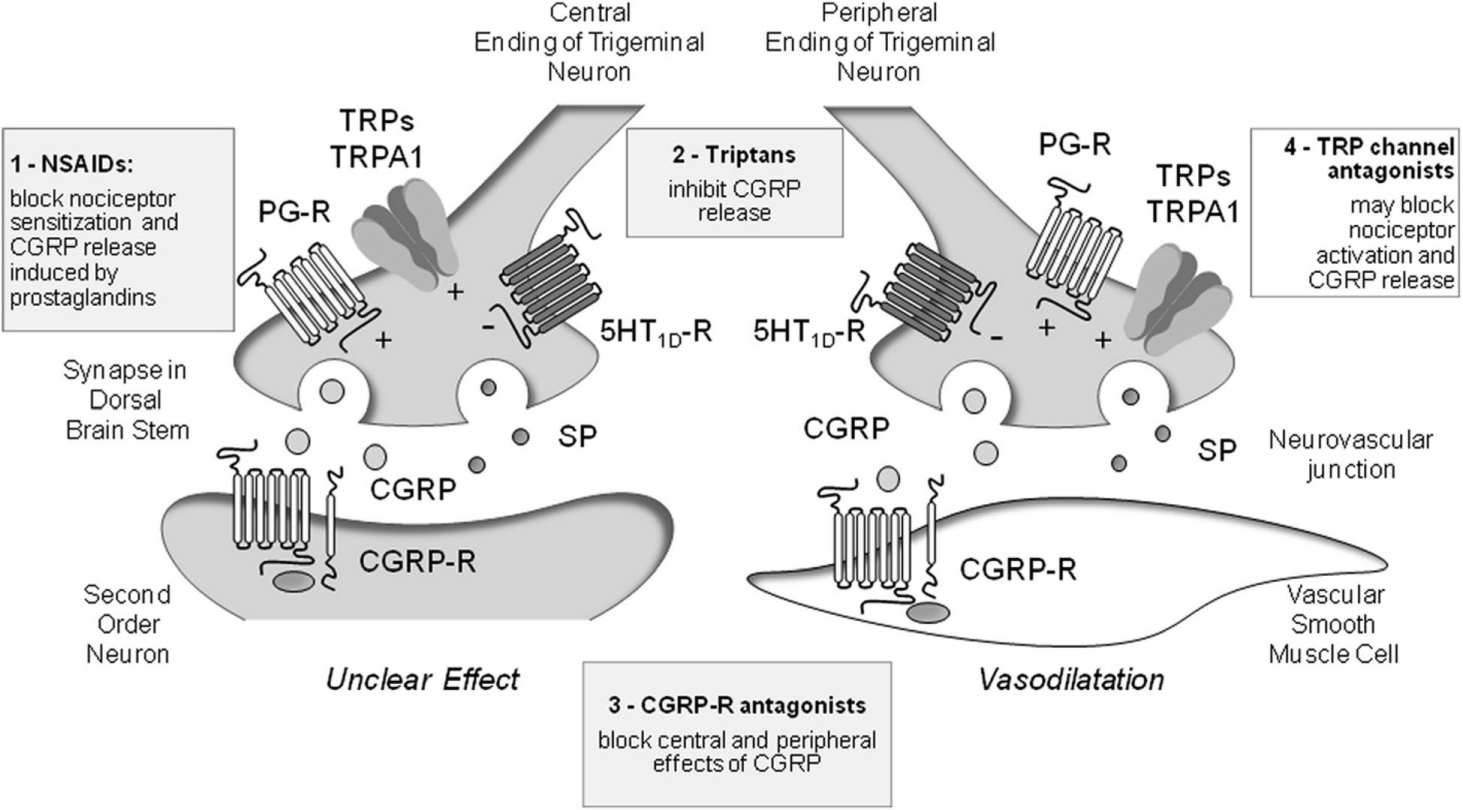
Mechanisms of action of antimigraine treatments used in chronic migraine and emerging treatments in relation to calcitonin gene-related peptide (CGRP). Modified with permission from Benemei, et al. J Headache Pain 2013;14:71 [29]

In patients with CM, acute treatment options, such as analgesics, non-steroidal anti-inflammatory drugs and triptans, should be reserved for clearly defined exacerbations of headache [8, 20, 36]. Opioid- and barbiturate-containing medication should also be avoided because of their strong association with MOH [12, 20]. Triptans are migraine-specific medications that inhibit the release of CGRP by activation of presynaptic 5HT1 receptors (Fig. 1) [29, 31, 37]. However, triptans are inappropriate for the treatment of CM because patients should not take them more often than 2 to 3 days per week to avoid developing MOH [8, 20, 31]. Effective acute treatment of migraine attacks with triptans may help to prevent progression from EM to CM, but rather than relying on taking drugs to stop migraine attacks after they have started, the aim of treatment for CM should be the prevention of migraine attacks [35].

### Current prophylactic treatment options

The first-line treatment of CM is pharmacological [35]. Although there is evidence in support of the use of non-invasive peripheral neurostimulation methods for the prevention of CM [38, 39], most neurostimulation-based and neuromodulatory treatment techniques need further investigation and should be reserved for the most challenging and intractable cases of CM [8, 35, 36]. Behavioural management techniques (e.g. cognitive therapy, exercise, stress management), alternative physical therapies (e.g. acupuncture) and nutraceutical therapies (e.g. supplementary magnesium, riboflavin and Coenzyme Q10) can all be used to complement pharmacological therapy [35, 40].

Numerous orally administered drugs are used for the prophylaxis of CM, including beta-blockers (propranolol, metoprolol), anticonvulsants (valproate, topiramate), calcium-channel blockers (flunarizine), tricyclic antidepressants (amitriptyline, nortriptyline), serotonin antagonists (pizotifen, methysergide), antihypertensives (lisinopril, candesartan), and antidepressants that act as serotonin norepinephrine reuptake inhibitors (venlafaxine), selective serotonin reuptake inhibitors (paroxetine, fluvoxamine) or noradrenergic and specific serotonergic antidepressants (mirtazapine) [41]. Treatments that are effective for EM are not necessarily effective for CM [11], but evidence for the efficacy of oral agents in CM is generally extrapolated from studies in patients with high-frequency EM [20, 36, 42]. Insufficient efficacy and/or adverse events leading to treatment discontinuation often occur with these drugs in patients with CM [43–45]. OnabotulinumtoxinA (OBT-A), which is a formulation of botulinum toxin A administered by intramuscular injection, and topiramate are the only currently available therapies with high-quality evidence for the preventive therapy of CM from more than one randomised controlled trial [20, 36, 42].

#### OnabotulinumtoxinA

To date, OBT-A is the only treatment specifically approved for the prevention of CM in the EU [46]. OBT-A has been available in Italy since 2013, where it has the highest level of recommendation for the prophylactic treatment of CM [41, 46, 47]. OBT-A has been shown to be an effective and generally well tolerated treatment for the prevention CM in the Phase III Research Evaluating Migraine Prophylaxis Therapy (PREEMPT) trials [48, 49], and tends to be better tolerated than various oral prophylactic treatments, including topiramate [50–53]. Based on the PREEMPT clinical trial paradigm, OBT-A is administered to at least 31 injection sites across 7 head and neck muscles, and is currently recommended as a second-line option for patients who have not responded adequately or are intolerant of commonly prescribed oral migraine treatments [47]. Treatment should be repeated every 3 months. It is thought that injection of OBT-A in the trigeminally-innervated cranio-facial-cervical region inhibits release of CGRP from peripheral nociceptive neurons and interferes with TRP channels (Fig. 1), thereby reducing neuronal hyperexcitability and peripheral and central sensitisation [11, 46]. It is hypothesised that trigeminal-targeted preventative treatments counteract the impingement of nociceptive input from highly sensitised trigeminal neurons on brain stem second order neurons, thus preventing central sensitisation, a key pathophysiological mechanisms of CM.

#### Topiramate

Like OBT-A, topiramate has the highest level of recommendation for the prophylactic treatment of CM in Italian treatment guidelines [41]. Although topiramate reduced headache days versus placebo and was relatively well tolerated in patients with CM in two large randomised controlled trials [54, 55], adverse events commonly associated with topiramate include paresthesia, memory and concentration disturbances, fatigue and nausea [41]. It is believed that topiramate is able to prevent the development of cortical spreading depression associated with migraine by modulating ion channels (e.g. blockade of voltage-gated sodium channels) and neurotransmitter release (e.g. inhibition of glutamate), resulting in inhibition of neuronal hyperexcitability [11, 32].

### Emerging prophylactic treatments targeting CGRP

There is still an unmet need for more effective, better tolerated prophylactic therapies aimed specifically at patients with CM or high-frequency EM [20]. CGRP and its receptor are well-validated targets for CM, and monoclonal antibodies against CGRP or its receptor are proving very promising for CM prophylaxis in clinical trials [56–58].

#### CGRP receptor antagonists

Small molecule CGRP receptor antagonists are thought to act by blocking the CGRP receptors in the central nervous system and peripheral tissues (Fig. 1), thereby inhibiting the physiological and cellular effects of CGRP [59]. Initially formulated for the acute management of migraine, CGRP receptor antagonists provided the proof of principle that targeting the CGRP pathway may effectively prevent migraine [27, 60]. Telcagepant has been investigated for the prevention of EM, and although CNS penetration is modest, randomised controlled trial data showed a reduction in headache days with telcagepant versus placebo [61]. However, the clinical development of telcagepant was discontinued because of hepatotoxicity concerns, and the development of several other CGRP receptor antagonists has also been discontinued because of safety concerns, formulation issues or unknown reasons [56]. Three CGRP receptor antagonists are currently in phase III development for migraine (atogepant, rimegepant, and ubrogepant), with atogepant and rimegepant being investigated as prophylactic treatment in EM [56, 62]; no studies of CGRP receptor antagonists have been conducted in patients with CM [56, 60].

#### Anti-CGRP/R monoclonal antibodies

Anti-CGRP antibodies are macromolecules that bind to the CGRP ligand or its receptor neutralising the effects of excessive CGRP released in the trigeminal sensory nerve fibres during migraine attacks (Fig. 1) [27, 60, 63]. Three anti-CGRP/R antibodies are approved in the US and Europe for the prophylactic treatment of CM: fremanezumab [64, 65] and galcanezumab [66, 67], which target the CGRP ligand; and erenumab [68, 69], which targets the CGRP receptor. A fourth anti-CGRP/R antibody against the CGRP ligand, eptinezumab, is currently under review by the US Food and Drug Administration [70]. These macromolecule anti-CGRP/R antibodies have been specifically designed for prophylactic use in CM and frequent EM, and to overcome safety issues associated with CGRP receptor antagonists [27, 57]. They are highly specific for their CGRP/R target, have no ability to cross the blood brain barrier, and bypass liver metabolism so CNS-related effects and hepatotoxicity are unlikely [71]. Erenumab, fremanezumab and galcanezumab are administered subcutaneously, and eptinezumab is administered intravenously [58]. Their long half-lives allow for dosing once a month, all four actives, or once every 3 months, for fremanezumab only [58, 60].

The results of two phase II trials and one phase III trial demonstrating the efficacy and safety of anti-CGRP/R monoclonal antibodies as prophylactic therapy in patients with CM have been published thus far [72–74]. In phase II trials, monthly injections of erenumab or fremanezumab for 3 months resulted in significant reductions in the primary endpoints of monthly migraine days and headache hours, respectively, versus placebo [63, 74]. In a randomised, double-blind phase III trial, monthly (675 mg dose at baseline and 225 mg at weeks 4 and 8) or quarterly (single 675 mg dose followed by placebo at weeks 4 and 8) injections of fremanezumab were similarly effective in significantly reducing both the average number of headache days/month and migraine days/month compared with placebo in the 12-week period after the first dose [73]. In relation to the number of headache days/month, a treatment effect was observed within 4 weeks after the initial dose. Anti-CGRP monoclonal antibodies were well tolerated in these trials. Mild to moderate injection site reactions were the most common treatment-related adverse events. Long-term safety data are not yet available. Fremanezumab is currently under review by the European Medicines Agency for the prophylactic treatment of chronic and episodic migraine [75, 76] . This review will be based on data from the pivotal phase III studies in these indications [73, 77].

## Chronic migraine management in Italy

On the basis of the results of a worldwide web-based survey, the mean total direct annual cost per CM patient (considering healthcare provider visits, hospitalisations, procedures and medications) in Italy was estimated to be €2648 versus €828 for EM [78]. Similarly, a study conducted in an Italian tertiary headache center reported that the total annual cost per CM patient was €2250 versus €523 for EM [79]. Most of the cost of CM is carried by the National Health Service (NHS) [79].

### Current problems

CM management in Italy is largely inadequate and expensive because of clinical and operational issues. Currently, patients with CM in Italy are managed at tertiary referral headache centres, but long waiting lists and the paucity of specialists at each center can hinder timely access to high-quality multidisciplinary care [80].

Given the heterogeneity of CM and debated ICHD clinical diagnostic criteria, patients with CM tend to be misdiagnosed [9, 81]. Patients with CM frequently undergo unnecessary procedures, such as electroencephalography and cervical spine imaging [81]. Furthermore, these expensive procedures are often repeated for no apparent reason other than inadequate traceability of their clinical history [81]. Problems diagnosing CM and different data collection instruments and strategies among headache centers mean that the true prevalence and cost of CM in Italy is not known.

It is also apparent that a high proportion of patients with CM do not receive prophylactic therapy in Italy [9, 10, 79]. In Italy, physicians may prescribe off-label treatment at their discretion after receiving informed consent from the patient, and many patients who are prescribed prophylactic medications are using drugs that are not evidence-based [9]. In its current definition, CM includes subgroups of patients with very different levels of severity and outcome [81, 82]. These subgroups are currently not recognised, and there is no strategy to tailor and optimise prophylactic therapy according to individual patient needs. As well as considering the frequency of migraine, effective individualisation of therapy would require careful assessment of clinical characteristics of migraine in each CM patient, as well as their overall medical history [82, 83]. In general, it seems likely that suboptimal use of potentially beneficial evidence-based prophylactic treatment is contributing to the high economic burden of CM in Italy.

### Moving towards a more rationalised and personalised treatment approach

The recent approval of OBT-A and promising phase II and III clinical trial results with anti-CGRP monoclonal antibodies for the prophylactic treatment of CM opens encouraging new therapeutic scenarios for CM management [81]. It prompts us to rethink our approach to CM in terms of customised healthcare, and to better define different CM phenotypes, endophenotypes and biological markers of response so as to facilitate best use of evidence-based prophylactic treatment options [81, 82]. There is an urgent need for a shared clinical and scientific management strategy that will shed light on neglected areas of clinical governance in relation to CM, minimise the risk of misdiagnosis, rationalise healthcare resource allocation and ensure that patients receive the treatment that best suits their clinical and personal needs. For example, establishing diagnostic and care plans, similar to the one implemented in Palermo for the management of pediatric headache [84], would provide a clear pathway through the diagnostic process, and would help to eliminate redundancy and unnecessary diagnostic procedures or treatments and channel appropriate patients to specialised care in multidisciplinary headache centers. These centers are best placed to identify patients with CM or EM that is trending towards chronicity, and particularly to make a differential diagnosis in patients with other conditions that can mimic CM. Ultimately, such a strategy would reduce the burden of CM on patients and the NHS.

The availability of a national CM register of CM will facilitate a move away from clinical empiricism towards precision medicine [85]. The Italian CM register now includes patient data from 28 headache centers and is soon to involve all Italian headache centers. The results of an exploratory pilot study of the Italian CM register have been published [81]. This study involved 63 consecutive patients with CM seen at four tertiary referral headache centers, where they were screened by specifically trained neurologists using a dedicated semi-structured questionnaire to gather information on variables such as lifestyle, behavioral and socio-demographic factors, comorbidities, migraine features before and after chronification (e.g. disease duration; location, quality and intensity of pain; attack duration and frequency; allodynia; unilateral cranial autonomic symptoms; previous acute and prophylactic treatments); and healthcare resource utilisation. The collected data revealed that most patients had symptoms linked to peripheral trigeminal activation (e.g. unilateral pain, pulsating quality, severe intensity). It was suggested that this simple clinical tool, using easily obtainable clinical details, could help to define different CM endophenotypes and predict responsiveness to topimarate, to OBT-A and anti-CGRP monoclonal antibodies [81]. In support of this, there is some evidence to suggest that OBT-A may be most effective in patients with higher interictal blood levels of CGRP and intense peripheral trigeminal activation [83, 86]. The database pilot study also uncovered neglected areas of clinical governance, such as inappropriate hospitalisations, procedures and medications, confirming the view that much work is needed to rationalise and optimise the management of CM in Italy.

### Creating a culture of prevention

An improvement in the use of appropriate prophylactic medication is clearly needed to reduce the burden of CM in Italy. The availability of effective and well tolerated new treatment strategies that are specifically indicated for the prophylaxis of CM may help to focus attention on preventive rather than acute treatment of migraine attacks in patients with CM. To this end, neurologists, general practitioners, pharmacists and patients all need to be well informed about CM and the new treatment options.

As observed with OBT-A in the PREEMPT clinical trial program [48], introduction of effective prophylactic therapy early after the onset of chronicity may result in greater benefits [47]. To make best use of prophylactic therapies such as OBT-A and anti-CGRP antibodies, it is therefore important to identify patients with CM and offer them prophylactic treatment as early as possible [47]. In order to identify patients with CM early in the course of the chronicity, patients with high-frequency EM should be monitored closely for headache frequency and new onset CM.

In addition to ensuring that patients with CM receive the best available prophylactic medication early after the onset of chronicity, prevention of chronification in patients with high-frequency EM would also help to limit the burden of CM and should be prioritised [87]. Modification of risk factors or the use of effective therapy has not been prospectively shown to prevent chronification, but it has been suggested that the risk of progression may potentially be reduced by a combined treatment approach of acute treatment to reduce migraine severity and prophylactic treatments to reduce migraine frequency [11]. A pooled analysis of clinical trial results suggests that prophylactic treatment with topiramate in patients with EM may help to prevent migraine chronification [88]. Anti-CGRP monoclonal antibodies may also prove to be useful in this regard, as suggested by the results of a phase II clinical trial in which fremanezumab significantly reduced migraine days versus placebo in patients with high-frequency migraine [72].

Although in this review we focus on the pharmacological prevention of CM, patient education, lifestyle factors, overuse of acute medication, and comorbidities all need to addressed in a multidisciplinary treatment plan to ensure optimal management of CM [20]. Patients should be well informed about CM and the treatment they are prescribed, and encouraged to take an active role in managing their condition by adopting positive lifestyle behaviors (e.g. regular sleep, meals and exercise routines), avoiding triggering and aggravating factors, and collaborating with their physician on a long-term treatment strategy [47].

## Final considerations

The recent introduction of OBT-A and positive phase II and III clinical trial results with anti-CGRP monoclonal antibodies for the prophylactic treatment of CM offers new hope for the many patients with CM who are currently not taking any prophylactic therapy or benefitting from their current treatment. In particular, monoclonal antibodies specifically targeting the CGRP pathway promise a major step forward for the prophylactic treatment of CM. However, to realise the full therapeutic potential of these drugs and effectively reduce the burden of CM in Italy, there is a need for increased disease awareness among both patients and physicians, more accurate diagnosis of CM and individualised evidence-based prophylactic treatment strategies.

However, we need to learn lessons from the past. The availability of triptans in 1990s created awareness about headaches and CM, but appropriate prescription of triptans by both general practitioners and specialists took a long time. Lessons from this time should inform our implementation of novel prophylactic agents for CM to ensure cost-effective use of these agents. The mechanism of action of these treatments is so specific to migraine that inappropriate use will almost invariably result in treatment failure, which in turn may make prescribers wary about the efficacy of these agents for patients who could actually benefit.

To reduce the risk of diagnostic error and avoid incorrect treatment practices with topiramate, OBT-A and anti-CGRP monoclonal antibodies, patients with CM should be managed at specialist headache centers, where they will receive a high level of multidisciplinary care. Going forward, patient profiles in Italy will be recorded on a national CM register, which will facilitate easy identification of patient subgroups who may respond to specific CM therapies. The national register will also support a shared patient management strategy among headache specialists.

Ideally, biochemical and clinical markers of therapeutic efficacy will be identified so that potential good responders to topiramate, OBT-A or anti-CGRP monoclonal antibodies can be targeted for treatment, thereby maximising the cost-effective use of these treatments. High interictal levels of CGRP and symptoms linked to peripheral trigeminal activation are possible candidates, but much research will be required before possible markers of therapeutic efficacy are used in routine clinical practice. The next phase of research should also aim to assess whether OBT-A and anti-CGRP monoclonal antibodies may also be used to prevent or delay transition of high-frequency EM to explicit CM.

## Conclusions

Significant advances are currently being made in the prophylactic treatment of CM, which open new and promising scenarios in CM management. These advances should prompt us to rethink the approach to this devastating disease and empower us to be vigilant and continue scientific clinical research, bearing in mind that only a common, shared clinical and scientific management strategy will improve CM ascertainment, distinguish phenotypes and biological markers, shed light on neglected clinical governance areas, provide customised healthcare and tailored therapy, optimise economic resources allocation and reduce the personal, social and economic burden of CM. It is up to us to ensure that newly established and emerging treatment options, such as OBT-A and the new anti-CGRP monoclonal antibodies, are used to their best effect within a wider culture of prevention so as to significantly reduce the personal, social and economic impact of this devastating disease.

## Data Availability

Not applicable

## Abbreviations

CGRP: Calcitonin gene-related peptide
CM: Chronic migraine
EM: Episodic migraine
ICHD-3: International Headache Society classification of Headache Disorders
MOH: Medication-overuse headache
NHS: National Health Service
OBT-A: OnabotulinumtoxinA
PREEMPT: Phase III Research Evaluating Migraine Prophylaxis Therapy
TRP: Transient receptor potential
